# The changing contribution of fentanyl use to non-fatal overdose among a cohort of people who inject drugs in San Diego, California: A longitudinal assessment

**DOI:** 10.1016/j.drugalcdep.2026.113106

**Published:** 2026-02-27

**Authors:** O.S. Jegede, D. Abramovitz, W.H. Eger, I. Artamonova, A.R. Bazzi, N.K. Martin, D. Werb, C.F. Vera, S.A. Strathdee, A. Bórquez

**Affiliations:** a Herbert Wertheim School of Public Health and Human Longevity Science, University of California San Diego, La Jolla, CA, USA; b School of Public Health Work, San Diego State University, San Diego, CA, USA; c School of Medicine, University of California San Diego, La Jolla, CA, USA; d Boston University School of Public Health, Boston, MA, USA; e Population Health Sciences, University of Bristol, Bristol, UK; f Centre on Drug Policy Evaluation, St. Michael’s Hospital, Toronto, ON, Canada; g School of Social Work, San Diego State University, USA

**Keywords:** Illicit drugs, People who inject drugs, Population attributable fraction, Overdoses

## Abstract

**Background:**

Fentanyl has driven overdoses across the U.S. in heterogeneous ways, with local variation in non-fatal overdoses remaining under-described. We sought to estimate the contributions of fentanyl to non-fatal overdose among people who inject drugs (PWID) in San Diego, CA, during a period of substantial drug supply evolution.

**Methods:**

Using convenience sampling, we recruited PWID living in San Diego into a longitudinal cohort study involving biannual assessments of opioid use and non-fatal overdose from 10/2020–05/2024. Multivariable Poisson regression assessed longitudinal associations between past six-month fentanyl use and non-fatal overdose risk. We calculated the confounder-adjusted longitudinal population attributable fraction (LPAF) to quantify fentanyl’s contribution to non-fatal overdoses over time.

**Results:**

Among 204 participants, the median age was 40.5 years, 73.0% were male, and 68.1% experienced homelessness. Fentanyl use increased from 51.0%(104/204) at Visit 1(10/2020–10/2021) to 62.4%(68/109) at Visit 6(10/2023–05/2024) while non-fentanyl opioid use decreased from 39.7%(81/204) to 5.5%(6/109). Past six-month non-fatal overdose decreased from 22.5%(46/204) to 15.6%(17/109) overall, and from 38.5%(40/104) to 19.1%(13/68) among those using fentanyl. Significant associations between fentanyl use and non-fatal overdose risk were observed at Visits 1 (Adjusted Relative Risk (ARR) 5.97, 95%CI: 2.37–15.00) and 5 (ARR 4.19, 95%CI: 1.17–15.00). The percentage of non-fatal overdoses contributed by fentanyl use in this cohort (LPAF) was 35%(0.35, 95%CI: 0.17–0.52).

**Conclusion:**

The contribution of fentanyl to non-fatal overdoses varied over time in our cohort. Disentangling the role of contextual factors such as opioid tolerance, fentanyl potency, and behavioral changes will support intervention design amid increasingly toxic drug supplies.

## 1. Introduction

People who inject drugs (PWID) in North America are at high risk of fatal and non-fatal opioid overdose due to the increasingly toxic unregulated drug supply (Centers for Disease Control and Prevention, 2023). In the United States (U.S.), the overdose crisis worsened markedly, rising from under 40,000 deaths in 2010, to approximately 70,000 deaths in 2017, to over 100,000 deaths per year between 2021 and 2023, with preliminary data suggesting a steep decline in 2024 (National Center for Health Statistics, 2025; Spencer et al., 2023). The exponential increase in fatal overdoses was accompanied by shifts in the unregulated drugs driving overdose mortality, particularly from fentanyl contamination of unregulated opioid (i.e., heroin) supplies (Meacham et al., 2020; Park et al., 2018). Fentanyl here denotes not only illicitly-manufactured fentanyl but also a wide range of fentanyl analogues, i.e., chemically modified derivatives that may vary substantially in potency and toxicity (Armenian et al., 2018).

Fentanyl use trends have shown significant geographic variation across the U.S. (Jalal et al., 2018) Though the Western U.S. was less impacted by fentanyl than the Eastern U.S. from 2013 to 2017 (Scholl et al., 2018), national reports of drug seizures show that the Western region accounted for the largest share of fentanyl seizures in 2023. Most of those seizures (about three-quarters) involved illegally manufactured fentanyl pills (Palamar et al., 2025). Drug seizure commonly serves as an indirect measure of substance availability and use trends (Palamar et al., 2021) and studies have documented correlations between fentanyl seizure intensity and overall overdose fatality rates nationally (Gladden et al., 2019; Zibbell et al., 2019, 2023), and rising drug overdose deaths in the Western region (National Center for Health Statistics, 2025).

Specifically, in 2022, the highest number of overdose deaths nationally was in California, which registered over 10,000 deaths, including 997 in San Diego County alone (Centers for Disease Control and Prevention, 2022, 2024). In the City of San Diego, fentanyl-related fatal overdoses increased from 45 in 2018 to 410 in 2022 (Gloria, 2023), representing an 811% increase over five years. Although a decrease in overdose death rates was observed in California during 2024 (National Center for Health Statistics, 2025) the situation remains dire with 8753 overdose deaths recorded between May 2024 and April 2025 (CDPH Substance and Addiction Prevention Branch, 2025).

Prior work along the San Diego–Tijuana corridor shows that the introduction of fentanyl reshaped risk environments for people who use drugs (PWUD) in ways tied to cross-border flows, policing, and market practices, rather than statewide or regional trends alone (Friedman et al., 2022). Consistent with this, border-proximate cohorts of PWID report overdose risks linked to (often unrecognized) fentanyl exposure in locally specific powder formulations (e.g., “China white”), underscoring supply heterogeneity even relative to counties further north (Bailey et al., 2022).

Non-fatal overdose is also of critical concern, as it is a strong predictor of subsequent fatal overdose (Caudarella et al., 2016; Goldman-Mellor et al., 2020). Existing research in San Diego has provided insights into the personal experiences and correlates of non-fatal opioid overdose among PWID (Bailey et al., 2022), highlighting concerns regarding the toxicity of local unregulated drug supplies, including increasing presence of fentanyl in methamphetamine (the primary drug used among PWID in San Diego), and shifting patterns in the type, frequency and mode of opioid use in this community (Bailey et al., 2022; Ivsins et al., 2020; Valasek et al., 2023). While prior studies have documented fentanyl’s central role in the overdose crisis and explored the experiences of non-fatal overdose among PWID (Bailey et al., 2022; Valasek et al., 2023), a critical gap remains in understanding how the changing patterns of fentanyl use and potency influence non-fatal overdose risk over time among populations at high risk, particularly in border-region contexts like San Diego, where the drug supply and use patterns evolve rapidly.

This study aimed to estimate the relative risk of non-fatal overdose associated with recent fentanyl use (i.e., reported use in the previous 6 months vs. not) in a cohort of PWID in San Diego, as well as the proportion of non-fatal overdoses that can be attributed to fentanyl over time in this cohort. To obtain these estimates, we analyzed data collected between October 2020 and April 2024 among a cohort of PWID residing in San Diego County. To our knowledge, this is among the first longitudinal cohort studies to quantify the changing overdose risk associated with fentanyl use and the changing contribution of fentanyl to overdose in this population. Considering fentanyl’s introduction into San Diego’s and the broader West Coast’s unregulated drug markets, understanding these dynamics is critical for informing the efforts of public health officials, frontline harm reduction providers, and clinicians working to mitigate overdose risk.

## 2. Methods

### 2.1. Sample and recruitment

This analysis used data from an ongoing longitudinal cohort study, *La Frontera*, which includes PWID from San Diego County, California, and Tijuana, Mexico (Strathdee et al., 2021). *La Frontera* was designed to characterize trends in the incidence of HIV, hepatitis C, and opioid overdose associated with binational drug markets and cross-border drug use between San Diego County and Tijuana.

To be eligible for participation, individuals had to have injected drugs in the past month (confirmed by injection stigmata, such as skin track marks, scars, and abscesses), be aged 18 years or older, be fluent in either English or Spanish, and reside in San Diego or Tijuana at baseline. In each city, a team of three to five bilingual outreach workers conducted recruitment on weekdays during varied times of day using a mobile van. Recruitment efforts targeted locations known for concentrated drug use in both cities, as described in prior publications (Bailey et al., 2023). Varying the recruitment time and diversifying the recruitment locations were implemented to minimize the bias that may enter a non-probability sample. Participants were recruited for baseline visits between October 2020 and 2021 and followed up semi-annually (Bailey et al., 2023). Participants underwent in-person interviewer--administered surveys, the details of which have been previously published (Strathdee et al., 2021). At the time of recruitment, 202 of the 612 study participants resided in Tijuana and 410 resided in San Diego.

Data used for this analysis were collected between October 2020 and May 2024 from study participants who completed baseline and at least one follow-up visit. Due to the specific context, unregulated drug supply, and timing of fentanyl penetration and access to harm reduction services in San Diego versus Tijuana, this analysis was limited to a subset of 204 participants who reported residing in San Diego at all their study visits (Valasek et al., 2023). The University of California San Diego and Xochicalco University institutional review boards reviewed and approved all study procedures.

### 2.2. Variables of interest

#### 2.2.1. Outcome variable

The outcome of interest, assessed at each study visit, was self-reported experience of non-fatal overdoses in the past six months. Participants were asked, “In the past six months, how many times have you overdosed?” Non-fatal overdose was defined as “a situation where you passed out, could not wake up, or your lips turned blue” following drug use. A response of zero was coded as ‘no overdose;’ any number greater than zero was coded as ‘having experienced at least one overdose.’

#### 2.2.2. Independent variables

Our independent variables of interest were a) self-reported fentanyl use in the past six months (yes vs. no) and b) time, a nominal variable representing when a study visit occurred. Self-reported fentanyl use included any method of use by participants (e.g., injecting, smoking, inhaling), including use of fentanyl by itself or fentanyl use in combination with other drugs. Time was categorized as follows: visit 1 (October 2020–October 2021), visit 2 (November 2021–April 2022), visit 3 (May 2022–October 2022), visit 4 (November 2022–April 2023), visit 5 (May 2023–October 2023), and visit 6 (November 2023–April 2024).

To describe participants’ trends of self-reported opioid use over time, a three-level categorical variable was created corresponding to: 1) any fentanyl use (fentanyl use by itself or in combination with other drugs), 2) other opioids but no fentanyl use (heroin, China white, oxycontin, or prescription opioids like Vicodin, Darvon, Percocet), and 3) no opioid use. “*China White”* was used to describe a white or off-white powder marketed as high-purity heroin.

#### 2.2.3. Covariates

Covariates were selected based on existing literature, theoretical relevance, and empirical evidence of associations with both fentanyl use and overdose risk (Bailey et al., 2022). Potential covariates included age in years, ethnicity (coded as ‘yes’ vs. ‘no’ for PWID from Hispanic, Latino, or Spanish origin), sex assigned at birth (male or female), race (White vs. non-White; Black vs. non-Black), marital status (married: yes vs. no), years of education completed, monthly income < 500 USD (yes vs. no), cross border drug use at recruitment (yes vs. no), and dichotomous experiences with homelessness, incarceration, arrest, casual sex, sex work, heroin use, cocaine use, methamphetamine use, injection drug use, receptive needle sharing, syringe services programs (SSP) use, and opioid agonist therapy (OAT) in the past six months. We also examined two continuous variables: safe injection self-efficacy and the average number of injections per day (Bailey et al., 2022; Chapman et al., 2021). The *safe injection self-efficacy* measure reflects participants’ confidence in engaging in safer injection practices (Bailey et al., 2024; Garfein et al., 2007). It is calculated as the mean of six items, each rated on a 4-point scale (1 = Absolutely sure I cannot, 2 = Pretty sure I cannot, 3 = Pretty sure I can, 4 = Absolutely sure I can). Higher scores indicate greater confidence in practicing safe injection behaviors.

Participants were classified as having experienced homelessness in the past six months if they primarily slept at locations such as a migrant worker camp, asylum seekers’ shelter, traditional shelter, welfare residence, vehicle (car, bus, truck, etc.), abandoned building, deportee shelter/camp, outdoors (streets, beach, canal, canyon), shooting gallery, other designated unsafe camping sites.

Participants were asked about the frequency of their incarceration in the past six months. A response of zero indicates no prior incarceration, while any number greater than zero signifies previous incarceration.

### 2.3. Statistical analyses

We described the analytic sample with respect to baseline characteristics, using frequencies and percentages for binary/categorical variables and median (IQR) for continuous variables. Descriptive statistics were calculated for the overall sample and also stratified by whether one reported experiencing at least an overdose during the study or not. We created line plots illustrating the proportion of participants by type of opioid use and the proportion experiencing non-fatal overdose in each group over time.

To guide covariate selection, we conducted univariate marginal (i.e., population-averaged) Poisson regressions with robust variance estimation for clustered data (Chen et al., 2018). We specified an unstructured covariance matrix to account for within-subject correlation induced by the repeated measures. The corresponding bivariate associations between each candidate covariate and the outcome were examined using a conservative significance threshold (α = 0.10). Variables meeting this criterion were considered for inclusion in multivariable models, with final selection informed by examination of correlations, associations with the primary predictor of interest, multicollinearity, and potential interactions. These bivariate tests were used solely to inform model building, and no inferential conclusions were drawn from them.

To evaluate the association between past six-month fentanyl use and non-fatal overdose, we used marginal multivariable Poisson regression with robust variance estimation. ^29^Just like in the univariate regressions, an unstructured covariance matrix was used to account for within-subject correlations. In the multivariable model, past 6 months fentanyl use (time-varying exposure) and study visit where included as the main predictors of interest, while cross-border drug use status at enrollment and incarceration within the previous six months (time-varying confounder) were included in the model as covariates as these two variables were determined to be potential confounders, based on their relationship with both, the overdose outcome and the exposure to fentanyl. Additionally, the interaction between the main predictors (i.e., fentanyl use and study visit) was included in the model, due to its statistical significance, suggesting that the association between fentanyl use and risk of overdose varied by study visit. Consequently, we evaluated the effect of fentanyl use on the outcome at each study visit and presented parameter estimates from the multivariable model corresponding to the effect of fentanyl on our primary outcome by study visit.

Next, we estimated the confounder-adjusted longitudinal population attributable fraction (LPAF), which we define as the percentage of non-fatal overdoses that would not have occurred in the source population (San Diego PWID) over the course of the study in the absence of fentanyl use. Unlike a traditional PAF, which is a snapshot in time, the LPAF considers how a risk factor’s impact evolves over a follow-up period in a cohort, taking into account changes in temporal factors, including patterns of fentanyl use, mode of administration, and potency, while accounting for other variables that may influence both the outcome and exposure, thereby capturing the potential contribution of fentanyl under dynamic, real-world conditions rather than assuming a single, static exposure level. To calculate the LPAF, we adapted some of the steps implemented by Lin et al. (2013), in their calculations of the “Longitudinal Extension of the Population Attributable Fractions (LE-AAF)”. Details of our LPAF calculations follow. First, we used the visit-specific risk ratios (RRs) from the multivariable Poisson regression model described above to estimate the visit-specific (i.e., corresponding to the 6-month interval prior to baseline and to each 6-month interval between study visits, respectively) confounder-adjusted attributable fractions of non-fatal overdose among those exposed to fentanyl (AF_e_) and the corresponding Population Attributable Fractions (PAFs), respectively. The AFe were calculated using the formula AF_e_ = ((RR-1)/RR) and the PAFs were calculated by using Miettinen’s formula (percent exposed to fentanyl among those reporting an overdose*AF_e_) (Miettinen, 1974). The visit-specific PAFs represent the percentage of non-fatal overdoses that would not have occurred in the source population during each 6-month interval in the absence of fentanyl use. Finally, we obtained the LPAF by calculating the weighted average of the visit-specific PAFs with person-time (months) spent at risk during each 6-month interval as the weights (Allore et al., 2015; Lin et al., 2013). More specifically, for participants who reported experiencing an overdose during the time interval between two study visits, their time spent at risk was calculated as half the interval between the two visits. For participants who did not experience an overdose during a corresponding interval, their time spent at risk was calculated as the length of the entire interval. Next, the time spent at risk during each 6-month interval was summed up across all participants to obtain the person-time spent at risk during the corresponding interval. Then, the visit-specific PAFs were multiplied by the corresponding person-time spent at risk to obtain the weighted visit-specific PAFs, which were then summed up and divided by the person-time over the entire duration of the study to obtain the weighted average of the visit-specific PAFs. The corresponding 95% confidence intervals for the AF_e_s, PAFs, and LPAF were constructed using the NLEstimate SAS macro, which allows for estimating linear or nonlinear combinations of parameters obtained from any model (given the model parameters and their variance-covariance matrix). We should note that we are referring to the AFs, PAFs, and the LPAF as being confounder-adjusted because we calculated these measures based on the RRs obtained from the multivariable Poisson model, which was adjusted for the aforementioned potential confounders.

To assess potential selection bias, we compared participants included in this study with the participants excluded (because they did not complete at least one follow-up) by using the Chi-Square or Fisher’s exact test for comparisons involving binary/categorical variables and Mann-Whitney tests for comparisons with continuous variables (see Supplementary Table 1).

Even though a relatively high percentage of participants completed at least one follow-up visit and were included in the analyses, loss to follow-up was considerable by the end of the study, necessitating careful evaluation in order to determine whether alternative analytical approaches were necessary to accommodate the missing data. We should note that our primary analytical method (i.e., Poisson regression via GEE) yields robust estimates if the data is Missing Completely at Random (MCAR). To gain insight into the mechanisms underlying loss to follow-up, we conducted supplementary analyses to assess whether attrition was associated with baseline exposure or outcome status. This assessment was carried out using two complementary approaches. The first approach examined whether there was an association between the missing data at each follow-up visit and the baseline risk of overdose and fentanyl exposure, respectively (see Supplementary Table 2a and 2b). The second approach consisted of conducting Little’s MCAR Chi-Square tests (see test results in the Supplementary). We did not find sufficient evidence to reject the MCAR hypothesis, suggesting that the estimates obtained from our analysis are likely unbiased.

For sensitivity analysis (see Supplementary Table 3), we explored the dataset that included both the study participants who resided in San Diego (which make up the analytical sample for our analysis) as well as participants who resided in Tijuana (who were not included in our analyses).

We conducted all data analyses using SAS v9.4 (SAS Institute, 2004), and created graphs using MS Excel.

## 3. Results

### 3.1. Participant characteristics

Among the 204 participants in our final analytic sample, the median age at baseline was 40.5 (Interquartile Range, IQR 34.0–53.0), and 73.0% identified as male. The median number of visits per participant was 3.0 (IQR 2.0–5.0).

Table 1 shows the baseline socio-demographic and behavioral characteristics of participants overall and stratified by whether they reported experiencing at least one non-fatal overdose during the study. Ninety-two participants experienced at least one overdose, while 112 participants did not experience any overdose over the course of the study. Additionally, 37 of the 204 participants (18%) experienced multiple overdoses during follow-up. At baseline, 78 participants (38.2%) reported never experiencing an overdose, 25 (12.3%) reported one prior overdose, and 101 (49.5%) reported multiple prior overdoses.

Participants who experienced at least one overdose were younger (38.0 years, IQR 32.5–49.5 vs. 43.0 years, IQR 34.5–54.0) and a smaller percentage identified as Black (4.3% vs. 15.2%). Additionally, a greater percentage of participants who experienced at least one overdose reported cross-border drug use at recruitment (23.9% vs. 12.5%), receptive needle sharing in the past six months (53.3% vs. 39.3%), and distributive needle sharing in the past six months (53.3% vs. 37.5%). The use of cocaine (31.5% vs. 16.1%), heroin (88.0% vs. 75.9%), and fentanyl (69.6% vs. 35.7%) in the past six months was also more commonly reported among participants who experienced at least one overdose (Table 1).

A total of 95 participants, representing 47% of the final analytic sample, were lost to follow-up between their last follow-up visit and the last study visit (i.e., visit 6). Overall, participants who were lost to follow-up were largely comparable in their socio-demographic characteristics to participants who remained in the study. Also, no statistically significant differences were observed between the two groups with respect to the exposure variable (opioid use; p = 0.200) or the outcome variable (non-fatal overdose in the past six months; p = 0.999) (Supplementary Table 1).

### 3.2. Trends in opioid use and past six-month non-fatal overdose experience

**Fig. 1.**
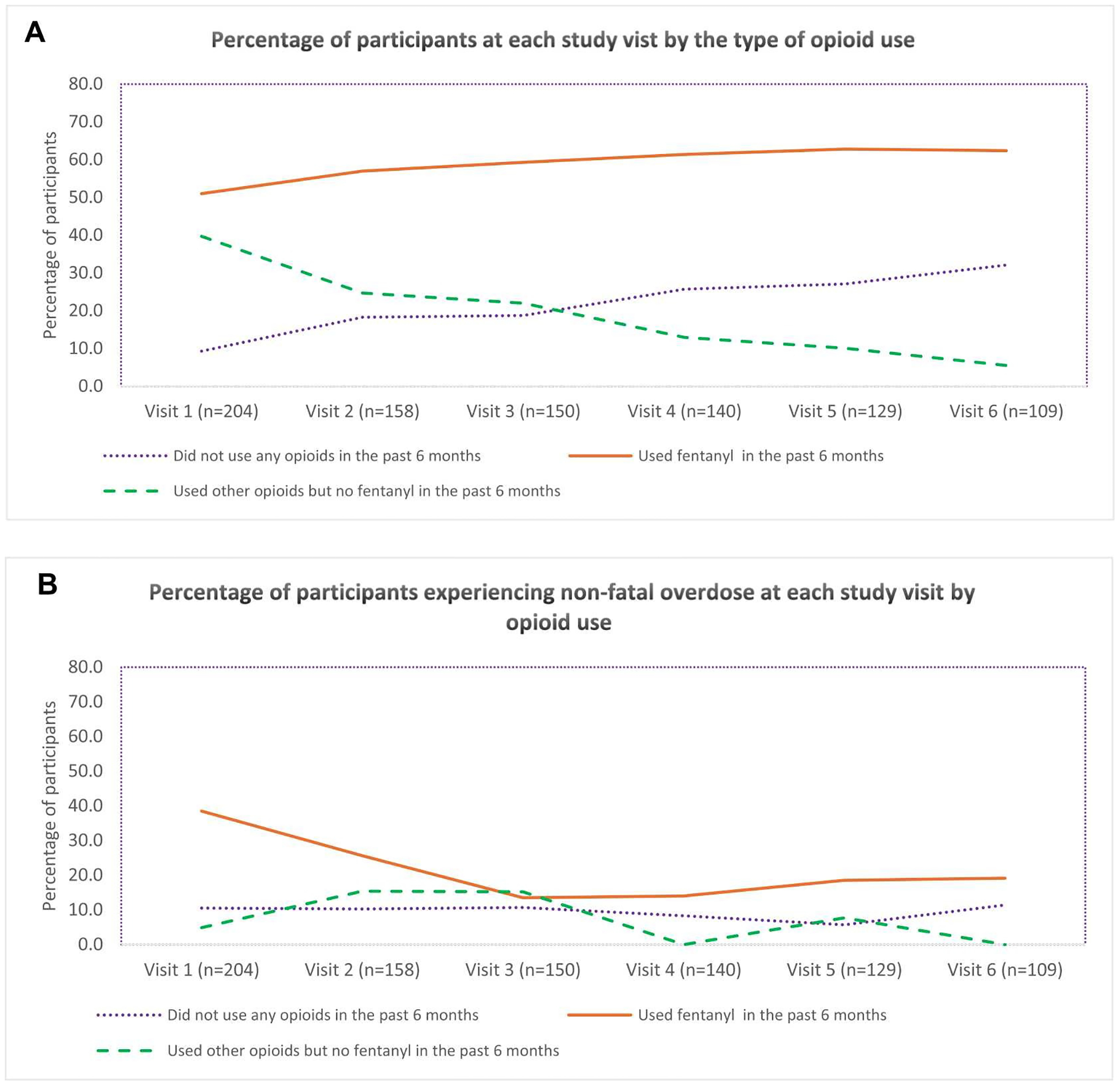

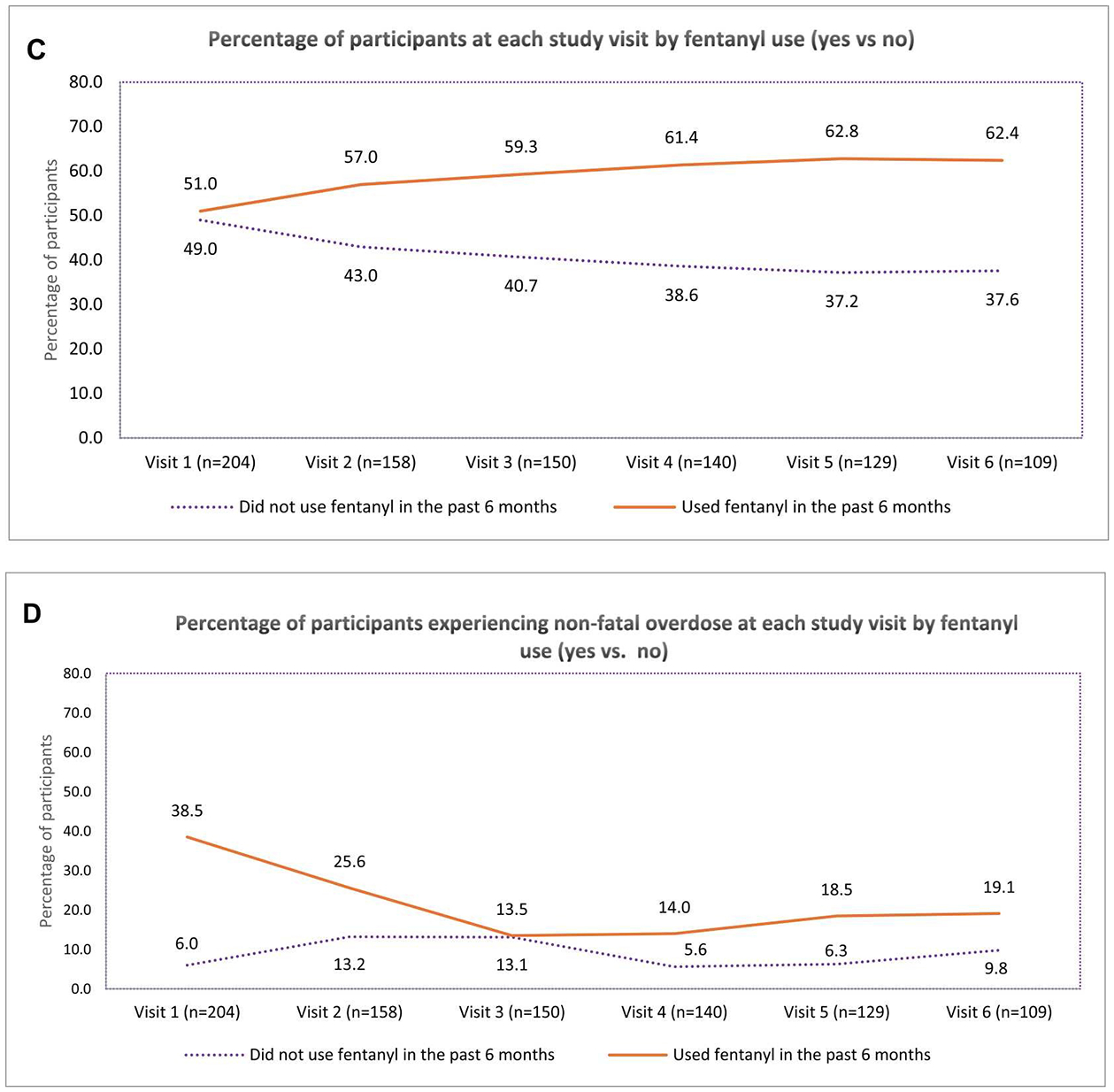
Plot of the percentage of participants at each study visit: A) by opioid use, B) experiencing non-fatal overdose by opioid use, C) by fentanyl use, D) experiencing non-fatal overdose by fentanyl use. *Visit 1,…, Visit 6 corresponds to the period between Oct 2020 and Oct 2021, Nov 2021 and April 2022, May 2022 and Oct 2022, Nov 2022 and April 2023, May 2023 and Oct 2023, Nov 2023 and April 2024, respectively.

Fig. 1A illustrates the percentage of participants at each study visit categorized by reported opioid use in the past six months. At baseline, 9.3% of participants did not use any opioids, 51.0% used fentanyl, and 39.7% used non-fentanyl opioids. By Visit 6, the proportion of participants who did not use any opioids was 32.1%, while those who used fentanyl increased to 62.4%. Additionally, those who used non-fentanyl opioids decreased to 5.5%.

Fig. 1B depicts the proportion of participants who reported experiencing non-fatal overdoses at each study visit, categorized by opioid use in the past six months. At baseline, 10.5% of participants who did not use any opioids experienced a non-fatal overdose, which increased to 11.4% by Visit 6. Among participants who used fentanyl, 38.5% experienced a non-fatal overdose at baseline, which decreased to 19.1% by Visit 6. For participants who used opioids other than fentanyl, 4.9% experienced a non-fatal overdose at baseline, which declined to 0.0% by Visit 6. The overall proportion recently experiencing a non-fatal overdose decreased from 22.5% at Visit 1 to 15.6% at Visit 6.

Fig. 1C shows the proportion of participants who reported using and not using fentanyl at each study visit (the latter combines the “other opioid use” and the “no opioid use” groups) and Fig. 1D shows the percentage of participants reporting experiencing non-fatal overdoses at each study visit, among each group. At Visit 1, 6.0% of participants who did not use fentanyl experienced an overdose; this percentage increased to 9.8% by Visit 6. Non-fatal overdose experience was consistently higher among those who used fentanyl, except for a nearly identical percentage at Visit 3.

Table 2 shows the unadjusted risk ratios for the association between fentanyl use, as well as other *a priori* selected variables, and non-fatal overdose over the study period. The crude risk ratio (RR) revealed a significantly higher overdose risk among individuals using fentanyl, which was 2.55 times that of those not using fentanyl (RR= 2.55, 95% CI: 1.69, 3.85).

### 3.3. Association between fentanyl use and past six-month non-fatal overdose experience

The relationship between fentanyl use and non-fatal overdose assessed via multivariable Poisson regressions at each visit, following a significant interaction between fentanyl use and study visit (p-value=0.004), was significant at Visit 1 (Adjusted RR = 5.97, 95% CI: 2.37, 15.00) and Visit 5 (Adjusted RR = 4.19, 95% CI: 1.17, 15.00) (Table 3).

### 3.4. Attributable fraction of non-fatal overdose due to fentanyl

Table 4 presents the confounder-adjusted attributable fractions of non-fatal overdose among those using fentanyl (AF_e_) and the confounder-adjusted population attributable fractions (PAFs). The AF_e_ among those exposed to fentanyl varied between 83% at Visit 1 and 64% at Visit 6, although confidence intervals were wide. Unlike at all the other visits, at Visit 3, the estimates consist of negative values, hinting at a possible inverse association between past six-month fentanyl use and non-fatal overdose at that time point. However, given the lack of statistical significance, this finding may be simply due to random sampling error. At Visit 1, 67% (PAF 0.67; 95% CI: 0.55, 0.80) of non-fatal overdoses among those who experienced an overdose could be attributed to fentanyl use. This implies that if fentanyl use were eliminated, we would expect a 67% reduction in the incidence of non-fatal overdose at Visit 1. At visits 5 and 6, respectively, 67% (PAF 0.67; 95% CI: 0.40, 0.94) and 49% (PAF 0.49; 95% CI: 0.17, 0.80) of non-fatal overdoses among those who experienced an overdose could be attributed to fentanyl use. The LPAF of fentanyl to non-fatal overdose over the entire observation period was 35% (LPAF 0.35; 95% CI: 0.17, 0.52), suggesting that 35% of non-fatal overdoses would have been averted in the absence of fentanyl.

## 4. Discussion

In this examination of opioid use patterns among a cohort of PWID in San Diego between October 2020 and May 2024, fentanyl use increased over the study period, accompanied by changes in the experience of non-fatal overdose. These trends underscore the growing dominance of fentanyl in the unregulated drug supply in the Western U.S and its changing effect on overdose risk (Centers for Disease Control and Prevention, 2015; United States Drug Enforcement Administration, n.d.). While prior studies have demonstrated an association between fentanyl use and overdose risk, our study adds a contribution by quantifying the attributable risk of non-fatal overdose due to fentanyl use in this population. By estimating both visit-specific and longitudinal PAFs, we provide new insight into the proportion of overdose events that could potentially be attributed to fentanyl, accounting for temporal trends and confounding.

At several time points during the study, over half of non-fatal overdoses were potentially attributable to fentanyl use, with PAFs reaching as high as 67%. The LPAF of 35% across the study period further underscores fentanyl’s substantial potential contribution to overdose risk.

Interestingly, despite the strong association of fentanyl use with non-fatal overdose, the proportion of PWID who reported experiencing non-fatal overdoses decreased over the study period despite reported increases in fentanyl use. The reduction in non-fatal overdoses aligns with recent estimates of decreasing fatal overdose rates in San Diego County (CDC Injury Center, 2023; County of San Diego Health and Human Services Agency Public Health Services Epidemiology and Immunization Services Branch, 2024). The observed decline in non-fatal overdoses could reflect a complex interplay of factors, including increased opioid tolerance over time, individual behavioral changes, both self-initiated and related to harm reduction efforts locally, and variations in the availability and purity of fentanyl and its analogues (Department of Public Health City of Philadelphia, 2022).

Individual-level opioid tolerance may partially explain the observed decline in non-fatal overdose risk over time. Repeated fentanyl use can lead to increased tolerance, as individuals’ bodies adapt to higher opioid levels (Allouche et al., 2014; Zhou et al., 2021). In terms of behavioral changes, we observed a decline in heroin and other opioid use, along with an increase in the proportion of individuals who ceased opioid use altogether. This shift may indicate a preference for non-opioid substances, such as methamphetamine (Eger et al., 2024), whose use has risen among people who previously used heroin due to its availability and perceived lower overdose risk (Palamar et al., 2020; Strickland et al., 2019, 2021). As awareness of fentanyl penetration in the unregulated drug supply increased in the U.S., many PWID transitioned from injecting to smoking to mitigate their risk of overdose (Abadie, 2023; Ciccarone et al., 2024a; Des Jarlais et al., 2007), which may partly explain why we observed a decrease in overdose risk over time. However, while the transition from injecting to smoking may coincide with reduced overdose risk (Institute for Public Health San Diego State University, 2022; San Diego County Health and Human Services Agency, 2023; San Diego Health and Human Services Agency, n.d.), evidence of a causal relationship is still unclear.

Paradoxically, non-fatal overdose risk was higher among those reporting no opioid use versus those reporting using heroin and non-fentanyl opioids. This may be driven by exposure to fentanyl-adulterated stimulants. A previous study has found that fentanyl is present in a notable proportion of non-opioid drug samples (12–15%), increasing the overdose risk for PWID who may not be aware of its presence (Wagner et al., 2023). Our study reinforces the growing concern regarding the widespread contamination of unregulated drugs with fentanyl and its association with a substantial increase in overdose risk (Daniulaityte et al., 2023; Lim et al., 2024; McKnight et al., 2023). Nonetheless, further research to understand the factors driving the observed higher overdose risk in this group, including qualitative inquiry, is needed to explain this finding.

Another lens to interpret the observed decline in non-fatal overdose rates is the “susceptibles” hypothesis, which posits that overdose risk may concentrate among a subpopulation of individuals particularly vulnerable to the toxic effects of opioids, such as those newly or infrequently using opioids, who have lower tolerance (Lim et al., 2022). As this group at higher risk experiences fatal overdoses or exits the opioid-using population, there may be a subsequent reduction in the pool of individuals most susceptible to overdose. This phenomenon could partially explain the observed decline in non-fatal overdoses over time, as the surviving population may increasingly consist of individuals with higher tolerance, greater experience navigating fentanyl in local drug markets, or enhanced engagement with harm reduction interventions.

In San Diego, the expansion of harm reduction initiatives, such as naloxone vending machines, increased syringe service programs, and enhanced drug overdose surveillance, may have also contributed to safer drug use behaviors and increased awareness of overdose risks in this cohort (Institute for Public Health San Diego State University, 2022; San Diego County Health and Human Services Agency, 2023; San Diego Health and Human Services Agency, n.d.). While naloxone does not prevent non-fatal overdoses, its availability, along with fentanyl test strips and other harm reduction tools, may have influenced the decline in non-fatal overdoses observed in this study by increasing the proportion of PWID aware and equipped with the knowledge and tools to prevent non-fatal overdoses (California Department of Health Care Services (DHCS), 2024; San Diego County Health and Human Services Agency, 2024). Yet, despite these advances, the slight rise in overdoses during later visits indicates the need for ongoing evaluation of harm reduction strategies and the broader dynamics of opioid use in San Diego.

### 4.1. Limitations and strengths

This study has several limitations. First, data collection for this study overlapped with the COVID-19 pandemic, a period marked by disruptions in drug supply and heightened social and economic stressors (Conway et al., 2022). These factors may have influenced substance use behaviors and overdose risk among participants during certain study waves and affected overall retention. Importantly, our findings were based on analyzing data from a non-probability, convenience sample with a relatively high degree of loss to follow-up by the end of the study. Even though extensive efforts were made by our team to recruit a sample representative of the target population by recruiting from a wide range of venues (mapped by our ethnographic team), during various days of the week and times of day, it is still possible that some selection bias might have entered the sample. Also, even though participants lost to follow-up were not found to differ from those included in the analyses with respect to key socio-demographics or baseline exposure to fentanyl or overdose risk, and even though we found no evidence of missing data not MCAR, there is still a chance that some attrition bias might have entered the sample. Furthermore, reliance on self-reported opioid use and experience of non-fatal overdoses may introduce recall, knowledge, and social desirability bias into the data. The actual substances consumed might not be fully captured, particularly as knowledge of drug supply contaminants may be inaccurate, and stigmatization of fentanyl may have changed over time. In particular, given that the study began in 2020, when awareness of fentanyl’s presence in drug markets in the western U.S. was still emerging, some participants may have underreported fentanyl use, especially if they were unaware of its presence in heroin or counterfeit pills at earlier study visits. These factors may lead to underreporting of fentanyl use in both early and later follow-up surveys. Finally, while our survey is comprehensive and we attempted to control for key confounders, unmeasured confounding remains a concern. As such, the RR estimates from our multivariable model and the measures based on these estimates (AFs, PAFs, and the LPAF), while expected to be robust based on the analytical methods used, may not be unbiased, and the stated limitations may compromise our ability to establish causality. Our findings should be interpreted with caution, especially if trying to make inferences beyond our target population (PWID residing in San Diego County), as they may not be generalizable to other geographic regions or to populations of PWUD who were not injecting at baseline.

Despite the limitations, this study is the first to examine and quantify the longitudinal relationship between fentanyl use and non-fatal overdose risk among PWID in San Diego County from October 2020 to April 2024, providing a more nuanced understanding of fentanyl’s contribution to the overall overdose epidemic in this population. Future research should explore how the transition from injection to smoking affects overdose risk, especially in light of our findings suggesting a weakening fentanyl-overdose association over time, potentially linked to a decline in injection frequency, as observed in other settings (Ciccarone et al., 2024b; Eger et al., 2024; Karandinos et al., 2024; Kral et al., 2021; Mars et al., 2024).

## 5. Conclusion

In conclusion, this longitudinal study of a cohort of PWID in San Diego County from October 2020 to April 2024 identified a rising prevalence of fentanyl use, which was associated with an increased non-fatal overdose risk. Contrary to expectations, the proportion of PWID reporting overdoses decreased over time, despite the significant association between fentanyl use and overdose. These findings provide nuanced insights into changing opioid trends in a cohort of PWID in San Diego and their potential implications for future research and public health interventions. The observed reduction in non-fatal overdose rates may be attributed to numerous factors, including the scale-up of harm reduction services across San Diego County, highlighting the importance of continuing to authorize and financially support harm reduction efforts to mitigate overdose risks among PWID in the context of an evolving unregulated drug supply.

## Supplementary Material

Supplementary Files

## Figures and Tables

**Table 1:** Baseline Characteristics of La Frontera, San Diego County-based, study participants by reported overdose (OD) experience between October 2020 and May 2024.

Baseline Characteristics^a^	Experienced at least one OD n=92	Did not experience any ODs n=112	Total n=204
Median Age (IQR)	38.0(32.5,49.5)	43.0(34.5,54.0)	40.5(34.0,53.0)
Sex assigned at birth (male)	71(77.2%)	78(69.6%)	149(73.0%)
Born in the US	89(96.7%)	107(95.5%)	196(96.1%)
Speaks English	91(98.9%)	110(98.2%)	201(98.5%)
Hispanic/Latino/Mexican	48(52.2%)	47(42.0%)	95(46.6%)
Racial group: White	60(65.2%)	59(52.7%)	119(58.3%)
Racial group: Black	4(4.3%)	17(15.2%)	21(10.3%)
Married or Common law	13(14.1%)	11(9.8%)	24(11.8%)
Monthly income <500 USD	39(42.4%)	35(31.3%)	74(36.3%)
Median # of years of education completed (IQR)	12.0(11.0,13.0)	12.0(10.5,12.0)	12.0(11.0,12.5)
Homeless*	65(70.7%)	74(66.1%)	139(68.1%)
Utilized SSP*	61(66.3%)	61(54.5%)	122(59.8%)
Cross border drug use at recruitment	22(23.9%)	14(12.5%)	36(17.6%)
Receptive needle sharing*	49(53.3%)	44(39.3%)	93(45.6%)
Distributive needle sharing*	49(53.3%)	42(37.5%)	91(44.6%)
Enrolled in OAT*	15(16.3%)	16(14.3%)	31(15.2%)
Spent time in jail or prison*^m^	19(20.9%)	10(8.9%)	29(14.3%)
Has/had a regular sex partner*^m^	31(34.1%)	33(29.5%)	64(31.5%)
Has/had casual sex partner(s)*^m^	32(35.2%)	29(25.9%)	61(30.0%)
Engaged in sex work*^m^	7(7.7%)	7(6.3%)	14(6.9%)
Smokes cigarettes	81(88.0%)	100(89.3%)	181(88.7%)
Median # of years of illegal drug use (IQR)	25.0(18.0,37.0)	28.5(20.0,39.5)	27.0(19.5,39.0)
Median # of years of injection drug use (IQR)	15.5(8.0,25.0)	20.5(11.0,32.5)	17.0(9.0,29.0)
Median self-efficacy score for safe injection (IQR)*	3.0(2.7, 4.0)	3.0(2.0, 3.7)	3.0(2.5, 3.7)
Median # of injections per day, on average (IQR)*	2.5(1.0, 4.0)	2.5(0.3, 2.5)	2.5(1.0, 3.3)
Used cocaine*	29(31.5%)	18(16.1%)	47(23.0%)
Used heroin*	81(88.0%)	85(75.9%)	166(81.4%)
Used methamphetamine*	84(91.3%)	98(87.5%)	182(89.2%)
Used fentanyl*	64(69.6%)	40(35.7%)	104(51.0%)
Used China White*	9(9.8%)	5(4.5%)	14(6.9%)
Opioids Use Group*			
Did not use any opioids	3(3.3%)	16(14.3%)	19(9.3%)
Used fentanyl	64(69.6%)	40(35.7%)	104(51.0%)
Used other opioids but no fentanyl	25(27.2%)	56(50.0%)	81(39.7%)
Tested HCV-seropositive^m^	56(60.9%)	52(46.8%)	108(53.2%)
Tested HIV-seropositive	4(4.3%)	5(4.5%)	9(4.4%)

a For the binary variables, the affirmative category is presented;

* Past six months; missing values

m n=1

**Table 2.** Association between selected variables and reported non-fatal overdose (over six longitudinal visits)^v^

Variable	Univariate RR (95% CI)
**Used fentanyl (yes vs. no)** *	**2.55 (1.69,3.85)**
**Cross border drug use at recruitment (yes vs. no)**	**1.90 (1.29,2.80)**
**Age (per one year increase)**	**0.99 (0.97,1.00)**
Sex at birth (male vs. female)	1.32 (0.86,2.03)
Born in the US (yes vs. no)	1.10 (0.37,3.31)
Speaks English (yes vs. no)	1.34 (0.26,6.89)
Hispanic/Latinx/Mexican vs. not	1.12 (0.78,1.60)
**Racial Group: White vs. not**	**1.40 (0.95,2.05)**
**Racial Group: Black vs. not**	**0.38 (0.14,1.06)**
Married (yes vs. no)	0.82 (0.47,1.43)
Monthly income<500 USD (yes vs. no)	1.07 (0.80,1.45)
Years of education completed	1.05 (0.97,1.15)
Homeless (yes vs. no)*	1.11 (0.75,1.62)
**Utilized Syringe service program (yes vs. no)** * ^m1^	**1.37 (0.97,1.93)**
**Receptive needle sharing (yes vs. no)** * ^m2^	**1.35 (0.97,1.87)**
Distributive needle sharing (yes vs. no)*^m2^	1.10 (0.79,1.53)
Enrolled in opioid agonist therapy (yes vs. no)*^m3^	1.17 (0.77,1.76)
**Spent time in jail or prison (yes vs. no)** * ^m5^	**2.48 (1.84,3.34)**
Has had a regular sex partner (yes vs. no)*^m6^	1.19 (0.86,1.63)
**Has had casual sex partners (yes vs. no)** * ^m7^	**1.45 (1.06,1.97)**
Engaged in sex work (yes vs. no) *^m8^	1.30 (0.76,2.21)
Smokes cigarettes (yes vs. no)	1.52 (0.84,2.75)
Injected any drugs (yes vs. no)*^m10^	1.38 (0.83,2.30)
# of years of illegal drug use (per one year increase)	0.99 (0.97,1.00)
**# of years of injection drug use (per one year increase)**	**0.99 (0.97,1.00)**
Self-efficacy score for safe injection (per one unit increase)^m9^	1.04 (0.83,1.30)
**# of injections per day on average (per one additional injection)** *	**1.16 (1.04,1.31)**
**Used cocaine (yes vs. no)** *	**1.73 (1.23,2.44)**
**Used heroin (yes vs. no)** *	**1.47 (1.00,2.14)**
**Used methamphetamine (yes vs. no)** *	**2.19 (1.32,3.64)**
**Used China White (yes vs. no)** *	**2.69 (1.91,3.78)**

v Visit was used as a main effect along with each variable listed in the table; parameters corresponding to visit not shown.

* Refers to the past six months.

m Number of missing observations (summed across visits): ^m1^n=11; ^m2^n=2; ^m3^n=4; ^m4^n=85; ^m5^n=4; ^m6^n=43; ^m7^n=46; ^m8^n=43; ^m9^n=27; ^m10^n=10; ^m11^n=17;

**Table 3.** Multivariable Poisson regression model of the association between fentanyl use and non-fatal overdose

	Non-fatal opioid overdose in the past six months	
Variable	Unadjusted RR (95% CI)	*Adjusted RR (95% CI)
Used fentanyl at Visit^λ^ 1 (yes vs. no)	**6.33 (2.82, 14.20)**	**5.97 (2.37, 15.00)** ^#1^
Used fentanyl at Visit 2	**1.96 (0.99, 3.86)**	1.54 (0.78, 3.04)
Used fentanyl at Visit 3	0.87 (0.45, 1.70)	0.74 (0.39, 1.40)
Used fentanyl at Visit 4	1.78 (0.71, 4.45)	1.36 (0.59, 3.14)
Used fentanyl at Visit 5	**3.84 (1.08, 13.7)**	**4.19 (1.17, 15.00)** ^#2^
Used fentanyl at Visit 6	2.03 (0.70, 5.95)	2.75 (0.87, 8.62)

* Model with interaction between fentanyl and visit, adjusted for cross-border drug use at recruitment, and spent time in jail or prison, using GEE and an unstructured correlation structure.

# *p*-values adjusted for multiple comparisons using the Benjamini–Hochberg (BH) procedure to control the false discovery rate at the 0.05 level. ^#1^p-value=0.0006; ^#2^p-value=0.08

λ Visit 1,…., Visit 6 corresponds to the period between Oct 2020 and Oct 2021, Nov 2021 and April 2022, May 2022 and Oct 2022, Nov 2022 and April 2023, May 2023 and Oct 2023, Nov 2023 and April 2024

**Table 4:** Visit-specific confounder-adjusted attributable fractions of non-fatal overdose among those exposed to fentanyl (AF_e_) and population attributable fractions (PAF) among all PWID (using the Miettinen’s formula)*

Variable	AFe (95% CI)	PAF (95% CI)
Visit 1^λ^	**0.83 (0.68, 0.98)**	**0.67 (0.55, 0.80)**
Visit 2	0.35 (−0.09, 0.79)	0.25 (−0.07, 0.57)
Visit 3	−0.34 (−1.21, 0.51)	−0.21 (−0.72, 0.31)
Visit 4	0.27 (−0.34, 0.87)	0.22 (−0.27, 0.70)
Visit 5	**0.76 (0.45. 1.06)**	**0.67 (0.40, 0.94)**
Visit 6	**0.64 (0.22, 1.05)**	**0.49 (0.17, 0.80)**

* Model was adjusted for cross-border drug use at recruitment and spent time in jail or prison.

λ Visit 1,…., Visit 6 corresponds to the period between Oct 2020 and Oct 2021, Nov 2021 and April 2022, May 2022 and Oct 2022, Nov 2022 and April 2023, May 2023 and Oct 2023, Nov 2023 and April 2024

## Appendix A. Supporting information

Supplementary data associated with this article can be found in the online version at doi:10.1016/j.drugalcdep.2026.113106.
