# Supplementary material for: The changing contribution of fentanyl use to non-fatal overdose among a cohort of people who inject drugs in San Diego, California: A longitudinal assessment
Source: Drug Alcohol Depend. Author manuscript; Available in PMC 2026 Apr 2. (PMC13041729; doi:10.1016/j.drugalcdep.2026.113106)
Supplement: Supplementary Files [file NIHMS2156414-supplement-Supplementary_Files.docx]

**THE CHANGING CONTRIBUTION OF FENTANYL USE TO NON-FATAL OVERDOSE AMONG A COHORT OF PEOPLE WHO INJECT DRUGS IN SAN DIEGO, CALIFORNIA: A LONGITUDINAL ASSESSMENT**

**SUPPLEMENTARY FILES**

Overall, participants excluded from the analysis because they did not complete at least one follow-up visit closely resembled those who remained in the study, though they differed on a few variables. Participants excluded were younger (median age 37.0 vs. 40.5 years; *p* = 0.021), reported fewer years of illicit drug use (23.0 vs. 27.0 years; *p* = 0.016), and had fewer experiences of homelessness (53.1% vs. 68.1%; *p* = 0.047) (***Supplementary Table 1***).

At baseline, the proportion of participants reporting at least one overdose in the past six months was nearly identical between those included and excluded from the analyses (22.5% [46/204] vs. 22.4% [11/49]). There were no statistically significant differences between participants excluded and those retained with respect to the exposure variable (opioid use group; *p* = 0.200) or the outcome variable (experienced overdose in the past 6 months; *p* = 0.999) (***Supplementary Table 1***).

***Supplementary Table 1. La Frontera San Diego Baseline Characteristics of PWID who completed at least one follow-up visit vs. not.***

| Baseline Characteristics^a^ | Did not complete a follow up visit^b^ N=49 | Completed at least one follow-up visit N=204 | Total N=253 | p |
| --- | --- | --- | --- | --- |
| Cross border drug use at recruitment | 13(26.5%) | 36(17.6%) | 49(19.4%) | .158 |
| Median Age (IQR) | 37.0(28.0,50.0) | 40.5(34.0,53.0) | 40.0(32.0,53.0) | .021 |
| Sex assigned at birth (male) | 38(77.6%) | 149(73.0%) | 187(73.9%) | .518 |
| Born in the US | 44(89.8%) | 196(96.1%) | 240(94.9%) | .139 |
| Speaks English | 48(98.0%) | 201(98.5%) | 249(98.4%) | .580 |
| Hispanic/Latino/Mexican | 18(36.7%) | 95(46.6%) | 113(44.7%) | .214 |
| Racial group: White | 25(51.0%) | 119(58.3%) | 144(56.9%) | .353 |
| Racial group: Black | 10(20.4%) | 21(10.3%) | 31(12.3%) | .053 |
| Married or Common law | 2(4.1%) | 24(11.8%) | 26(10.3%) | .186 |
| Monthly income <500 USD | 17(34.7%) | 74(36.3%) | 91(36.0%) | .836 |
| Median # of years of education completed (IQR) | 12.0(11.0,12.0) | 12.0(11.0,12.5) | 12.0(11.0,12.0) | .835 |
| Homeless* | 26(53.1%) | 139(68.1%) | 165(65.2%) | .047 |
| Utilized SSP* | 23(46.9%) | 122(59.8%) | 145(57.3%) | .102 |
| Receptive needle sharing* | 19(38.8%) | 93(45.6%) | 112(44.3%) | .389 |
| Distributive needle sharing* | 18(36.7%) | 91(44.6%) | 109(43.1%) | .318 |
| Enrolled in OAT* | 9(18.4%) | 31(15.2%) | 40(15.8%) | .585 |
| Spent time in jail or prison*^m1^ | 9(18.8%) | 29(14.3%) | 38(15.1%) | .438 |
| Has/had a regular sex partner*^m1^ | 12(25.0%) | 64(31.5%) | 76(30.3%) | .376 |
| Has/had casual sex partner(s)*^m1^ | 20(41.7%) | 61(30.0%) | 81(32.3%) | .122 |
| Engaged in sex work*^m1^ | 4(8.3%) | 14(6.9%) | 18(7.2%) | .756 |
| Smokes cigarettes | 42(85.7%) | 181(88.7%) | 223(88.1%) | .558 |
| Median # of years of illegal drug use (IQR) | 23.0(13.0,33.0) | 27.0(19.5,39.0) | 26.0(18.0,38.0) | .016 |
| Median # of years of injection drug use (IQR) | 12.0(8.0,28.0) | 17.0(9.0,29.0) | 17.0(9.0,29.0) | .133 |
| Median self-efficacy score for safe injection(IQR)* | 3.0(2.5, 3.8) | 3.0(2.5, 3.7) | 3.0(2.5, 3.7) | .811 |
| Median # of injections per day, on average (IQR)* | 2.5(0.3, 4.0) | 2.5(1.0, 3.3) | 2.5(0.7, 4.0) | .441 |
| Used cocaine* | 11(22.4%) | 47(23.0%) | 58(22.9%) | .930 |
| Used heroin* | 43(87.8%) | 166(81.4%) | 209(82.6%) | .290 |
| Used methamphetamine* | 46(93.9%) | 182(89.2%) | 228(90.1%) | .430 |
| Used fentanyl* | 21(42.9%) | 104(51.0%) | 125(49.4%) | .307 |
| Used China White* | 7(14.3%) | 14(6.9%) | 21(8.3%) | .091 |
| Opioids Use Group* |  |  |  | .200 |
| Did not use any opioids | 2(4.1%) | 19(9.3%) | 21(8.3%) |  |
| Used fentanyl | 21(42.9%) | 104(51.0%) | 125(49.4%) |  |
| Used other opioids but no fentanyl | 26(53.1%) | 81(39.7%) | 107(42.3%) |  |
| Overdose in the past 6 months | 11 (22.4%) | 46 (22.5%) | 57 (22.5%) | .990 |
| Tested HCV-seropositive^m1^ | 19(38.8%) | 108(53.2%) | 127(50.4%) | .070 |
| Tested HIV-seropositive | 1(2.0%) | 9(4.4%) | 10(4.0%) | .692 |

^a^For the binary variables, the affirmative category is presented.

^b^Of the 49 study participants who did not complete follow-up visits, 8 were reported to have died, confirmed by obituary or county records. Overdose was not listed as the cause of death for any. Among the 204 participants who completed at least one follow-up visit and were included in the analytic sample, five died from overdose-related causes.

*Past 6 months; missing values ^m^n=1

The level of attrition by the end of the study was substantial and warrants careful consideration. To better understand the loss-to-follow-up mechanism, we conducted additional analyses to assess whether attrition was related to the outcome or exposure at baseline. We approached this in two ways.

First, for each follow-up visit, we created indicator variables to represent missing data. We then examined Spearman correlations between these missing-data indicators and baseline variables for overdose and fentanyl use. The correlation coefficients for overdose ranged from –0.02 to 0.06 (***Supplementary Table 2a***), and for fentanyl use from –0.02 to –0.08 (***Supplementary Table 2b***). None of these correlations approached statistical significance, indicating that dropout was unlikely to be related to either the outcome or the exposure. These findings suggest that the missing data mechanism was independent of both the outcome variable and the exposure.

Second, we conducted chi-square tests to assess whether the data were missing completely at random (MCAR), following the approach described by Little (1998, Journal of the American Statistical Association).^1^ For the outcome variable (overdose in the past 6 months), the chi-square statistic was 366.5 (p = 0.093), and for the exposure variable (fentanyl use in the past 6 months), the corresponding values were 344.6 (p = 0.305). These results cautiously suggest that there is insufficient evidence to reject the MCAR hypothesis, indicating that the estimates obtained in our analysis are unlikely to be biased due to missing data.

***Supplementary Table 2a. Spearman correlation between participants missing data at visit 2 to 6 and experiencing overdose at baseline***

| \|  \| **Spearman Correlation Coefficients, N = 253 (Prob > \|r\| under H₀: Rho = 0)** \| \| \| --- \| --- \| --- \| \| **Variable** \| **(Overdose, past 6 months, reported at baseline)** \|  \| \| **Visit 2** \|  \|  \| \| Missing overdose data \| 0.03120 (p = 0.6214) \|  \| \| **Visit 3** \|  \|  \| \| Missing overdose data \| 0.03456 (p = 0.5843) \|  \| \| **Visit 4** \|  \|  \| \| Missing overdose data \| –0.02776 (p = 0.6604) \|  \| \| **Visit 5** \|  \|  \| \| Missing overdose data \| 0.05797 (p = 0.3584) \|  \| \| **Visit 6** \|  \|  \| \| Missing overdose data \| 0.02975 (p = 0.6376) \|  \| |
| --- | --- | --- | --- | --- | --- | --- | --- | --- | --- | --- | --- | --- | --- | --- | --- | --- | --- | --- | --- | --- | --- | --- | --- | --- | --- | --- | --- | --- | --- | --- | --- | --- | --- | --- | --- | --- |

***Supplementary Table 2b. Spearman correlation between participants missing data at visits 2 to 6 and fentanyl use at baseline***

| \|  \| **Spearman Correlation Coefficients, N = 253 Prob > \|r\| under H₀: Rho = 0** \| \| \| --- \| --- \| --- \| \| **Variable** \| **(Fentanyl Use, past 6 months, reported at baseline)** \|  \| \| **Visit 2** \|  \|  \| \| Missing fentanyl data \| –0.06427 (p = 0.3085) \|  \| \| **Visit 3** \|  \|  \| \| Missing fentanyl data \| –0.07868 (p = 0.2123) \|  \| \| **Visit 4** \|  \|  \| \| Missing fentanyl data \| –0.04500 (p = 0.4761) \|  \| \| **Visit 5** \|  \|  \| \| Missing fentanyl data \| –0.02000 (p = 0.7515) \|  \| \| **Visit 6** \|  \|  \| \| Missing fentanyl data \| –0.06619 (p = 0.2943) \|  \| |
| --- | --- | --- | --- | --- | --- | --- | --- | --- | --- | --- | --- | --- | --- | --- | --- | --- | --- | --- | --- | --- | --- | --- | --- | --- | --- | --- | --- | --- | --- | --- | --- | --- | --- | --- | --- | --- |

***Sensitivity analysis including Tijuana participants in the multivariable model of the association between fentanyl use and non-fatal overdose.***

For sensitivity analysis (see Supplementary Table 3), we explored the dataset that included both the study participants who resided in San Diego (which make up the analytical sample for our analysis) as well as participants who resided in Tijuana (who were not included in our analyses). A statistically significant three-way interaction between visit, participant’s city of residence, and fentanyl use suggested that the analyses evaluating the effect of fentanyl use on overdose should be stratified by participant residence. However, stratified analysis for participants residing in Tijuana was not feasible due to sparse overdose data, particularly at visits 4, 5, and 6. This is the reason why this paper focused on evaluating the effect of fentanyl use on overdose only among PWID residing in San Diego. Nevertheless, if we were to ignore the three-way interaction and fit a multivariable model with pooled data from both locations, including the same covariates as the original model, with participant residence replacing cross-border drug use and incorporating the interaction between visit and fentanyl use, the corresponding findings do not contradict the findings presented in this paper

***Supplementary Table 3. Multivariable model of the association between fentanyl use and non-fatal overdose using pooled data from San Diego and Tijuana.***

| **Variable** | **Adjusted RR** | **95% LCL** | **95% UCL** | **Chi-Square** | **Pr>ChiSq** |
| --- | --- | --- | --- | --- | --- |
| Used fentanyl (yes vs. no) at visit 1 | 2.9553 | 1.9548 | 4.4678 | 26.41 | <.0001 |
| Used fentanyl (yes vs. no) at visit 2 | 1.2251 | 0.7883 | 1.9040 | 0.81 | 0.3669 |
| Used fentanyl (yes vs. no) at visit 3 | 1.6769 | 0.8992 | 3.1271 | 2.64 | 0.1040 |
| Used fentanyl (yes vs. no) at visit 4 | 5.3879 | 1.9680 | 14.7508 | 10.74 | 0.0010 |
| Used fentanyl (yes vs. no) at visit 5 | 6.7446 | 2.1968 | 20.7073 | 11.12 | 0.0009 |
| Used fentanyl (yes vs. no) at visit 6 | 3.0436 | 1.0216 | 9.0682 | 3.99 | 0.0457 |

****The model used the same covariates as the primary analysis, but replaced cross-border drug use (CBDU) with participant residence and incorporated the interaction between visit and fentanyl use.***

**References:**

1. Little RJA. A test of missing completely at random for multivariate data with missing values. J Am Stat Assoc [Internet]. 1988 [cited 2025 Oct 22];83(404):1198–202. Available from: https://scholar.google.com/scholar_url?url=https://www.tandfonline.com/doi/pdf/10.1080/01621459.1988.10478722%3Fcasa_token%3DVSnWDqlNxZYAAAAA:XtapU5ACoouea4Wz8ZKLdCJrsvpkGqS2L34CW-RYhyWg_yqTgEc-XpcExNOY_jURb5XAmwbQJg&hl=en&sa=T&oi=ucasa&ct=ucasa&ei=pQn7aLaZE9rZzwKX7-SYDw&scisig=ABGrvjK-FdQG7QFjEBlMOVp9LJmF
